# Identification of key lipid metabolism-related genes in Alzheimer’s disease

**DOI:** 10.1186/s12944-023-01918-9

**Published:** 2023-09-22

**Authors:** Youjie Zeng, Si Cao, Nannan Li, Juan Tang, Guoxin Lin

**Affiliations:** 1 Department of Anesthesiology, Third Xiangya Hospital, Central South University, Changsha, 410013 Hunan China; 2 Department of Nephrology, Third Xiangya Hospital, Central South University, Changsha, 410013 Hunan China

**Keywords:** Alzheimer's disease, Bioinformatics, Biomarkers, Lipid metabolism, Differentially expressed genes, Differential expression analysis, Hub genes, Immune cell infiltration, Key genes

## Abstract

**Background:**

Alzheimer’s disease (AD) represents profound degenerative conditions of the brain that cause significant deterioration in memory and cognitive function. Despite extensive research on the significant contribution of lipid metabolism to AD progression, the precise mechanisms remain incompletely understood. Hence, this study aimed to identify key differentially expressed lipid metabolism-related genes (DELMRGs) in AD progression.

**Methods:**

Comprehensive analyses were performed to determine key DELMRGs in AD compared to controls in GSE122063 dataset from Gene Expression Omnibus. Additionally, the ssGSEA algorithm was utilized for estimating immune cell levels. Subsequently, correlations between key DELMRGs and each immune cell were calculated specifically in AD samples. The key DELMRGs expression levels were validated via two external datasets. Furthermore, gene set enrichment analysis (GSEA) was utilized for deriving associated pathways of key DELMRGs. Additionally, miRNA-TF regulatory networks of the key DELMRGs were constructed using the miRDB, NetworkAnalyst 3.0, and Cytoscape software. Finally, based on key DELMRGs, AD samples were further segmented into two subclusters via consensus clustering, and immune cell patterns and pathway differences between the two subclusters were examined.

**Results:**

Seventy up-regulated and 100 down-regulated DELMRGs were identified. Subsequently, three key DELMRGs (DLD, PLPP2, and PLAAT4) were determined utilizing three algorithms [(i) LASSO, (ii) SVM-RFE, and (iii) random forest]. Specifically, PLPP2 and PLAAT4 were up-regulated, while DLD exhibited downregulation in AD cerebral cortex tissue. This was validated in two separate external datasets (GSE132903 and GSE33000). The AD group exhibited significantly altered immune cell composition compared to controls. In addition, GSEA identified various pathways commonly associated with three key DELMRGs. Moreover, the regulatory network of miRNA-TF for key DELMRGs was established. Finally, significant differences in immune cell levels and several pathways were identified between the two subclusters.

**Conclusion:**

This study identified DLD, PLPP2, and PLAAT4 as key DELMRGs in AD progression, providing novel insights for AD prevention/treatment.

**Supplementary Information:**

The online version contains supplementary material available at 10.1186/s12944-023-01918-9.

## Introduction

Alzheimer’s disease (AD) represents a common neurodegenerative condition linked to aging process, resulting in gradual and degenerative dementia [1]. Approximately 5.8 million individuals suffered from AD In the United States in 2020, and this number could rise to 13.8 million by 2050 [2]. The characteristic pathological characteristics of AD include the manifestation of β-amyloid (Aβ) plaques and the accumulation of neurofibrillary tangles [3]. Previous research has investigated changes in in vivo biomarkers using cerebrospinal fluid (CSF) analysis [4], neuroimaging [5], and gene mutation analysis [6, 7]. Nevertheless, the understanding of the metabolic foundation of AD is still limited, and the connection between metabolic irregularities and the development of AD is yet to be determined.

Lipid metabolism is a multifaceted process, encompassing the catabolism, anabolism, and translocation of lipids within the organism. Lipids are indispensable for the typical development and functionality of the CNS. Notably, sphingolipids and cholesterol, being the primary lipid types, are primarily situated within the myelin of the CNS [8]. Impaired lipid metabolism within the brain is considered a pivotal factor contributing to the pathogenesis of neurodegenerative diseases [9, 10]. Plasma phospholipid concentrations have been linked to cognitive impairment in AD patients [11], with notable changes in sphingomyelin and ceramide being identified during initial stages [12]. A recent investigation unveiled a strong correlation between 26 metabolites, including sphingolipids, and hippocampal atrophy in conjunction with biomarkers linked to AD pathology [13]. Moreover, cholesterol accumulation was detected in affected brain regions of AD patients [14] and in relation to region-specific synaptic deficiencies [15]. These findings reveal a strong relationship between lipid metabolism and AD, although the precise molecular mechanisms remain elusive.

This study aimed to identify key differentially expressed lipid metabolism-related genes (DELMRGs) related to AD progression by conducting a comprehensive analysis using public datasets. Additionally, immune infiltration analysis was conducted to assess the correlation between key DELMRGs and immune cells specifically in AD samples. Moreover, the potential functions of key DELMRGs were assessed using gene set enrichment analysis (GSEA). Furthermore, a potential transcription factor (TF)-miRNA regulatory network of the key DELMRGs was predicted. Finally, based on the DELMRGs, AD samples were divided into two subclusters, and the differences in immune cells and pathways between subclusters were examined.

## Methods

### Study design

**Fig. 1:**
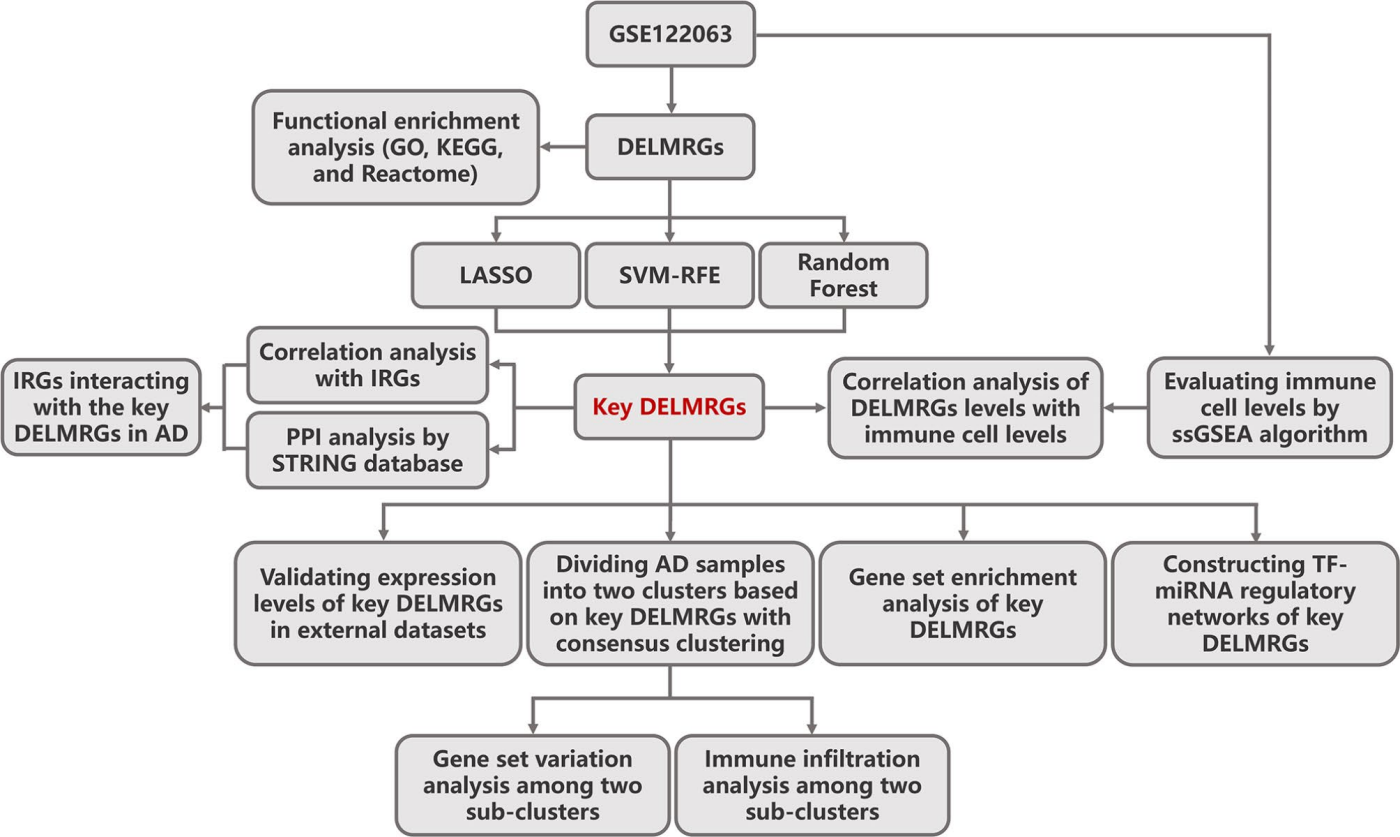
General flow chart

The general flow chart is presented in Fig. 1. Initially, the exploratory dataset GSE122063 was used to identify DELMRGs in AD brain tissues. Afterward, the DELMRGs underwent functional enrichment analysis. Next, key DELMRGs were identified by using three diverse algorithms. Then, correlations between key DELMRGs and immune cells were assessed. Notably, validation for these key DELMRGs was conducted in external datasets. Furthermore, GSEA was utilized to investigate the possible roles of the key DELMRGs. In addition, a regulatory network involving TF-miRNA was established for key DELMRGs. Moreover, within the validation dataset GSE33000, AD samples were segregated into two subclusters using consensus clustering, based on key DELMRGs. Subsequently, immune cell patterns and pathway differences between the two subclusters were examined. Finally, a correlation analysis of DELMRGs and immune-related genes (IRGs) was conducted, followed by a protein-protein interaction (PPI) analysis, and these analyses successfully identified IRGs that exhibit strong correlations and interactions with key DELMRGs in AD.

### Datasets acquisition

The datasets were derived from the Gene Expression Omnibus (GEO) platform [16]. Specifically, three microarray datasets for gene transcript expression levels were included. GSE122063 was an exploratory dataset used to identify key DELMRGs between the AD group and controls. To validate the expression levels of key DELMRGs between groups, GSE132903 and GSE33000 were utilized as validation datasets. Supplementary Table S1 provides comprehensive details about these datasets.

### Identification of DELMRGs between AD and control groups

The gene sets for lipid metabolism were downloaded from the MSigDB database [17]. Following that, 867 distinct lipid metabolism-related genes (LMRGs) were identified (Supplementary Table S2). Next, by “limma” R package, differentially expressed genes (DEGs) in GSE122063 were identified [18]. Based on previous studies [19, 20], |log_2_ fold change| > 0.5 and false discovery rate (FDR) < 0.05 was set as the threshold for detecting DEGs. This relatively lenient threshold was used to identify more potentially promising results. The overlapping genes between DEGs and LMRGs were identified as DELMRGs. Finally, the DELMRGs were visualized using a circular heatmap via the “circlize” R package [21].

### Functional enrichment analysis of DELMRGs

Functional enrichment analyses for DELMRGs were implemented in the DAVID database [22]. The analyses encompassed Gene Ontology (GO) [23], Kyoto Encyclopedia of Genes and Genomes pathway (KEGG) [24], and Reactome pathway enrichment analyses [25]. Subsequently, the Sangerbox online platform was utilized to visualize dot plots for the top 10 enriched terms (*P* < 0.05) [26].

### Screening of AD key DELMRGs

Subsequently, three machine-learning approaches [(i) least absolute shrinkage and selection operator (LASSO), (ii) support vector machine recursive feature elimination (SVM-RFE), (iii) random forest] were utilized for further determining key DEMLRGs. The LASSO algorithm was employed to minimize regression coefficients and eliminate redundant and uncorrelated genes from the analyses, reducing the risk of overfitting [27]. The SVM-RFE algorithm, based on support vector machines, is frequently utilized for optimal gene selection by minimizing classification errors and avoiding overfitting since the most accurate gene features could be filtered at the minimum root-mean-square error (RMSE) [28]. Random forest is a powerful machine-learning algorithm for gene selection in microarray analysis that is known for its robustness, ability to handle noisy and high-dimensional data, and accurate variable importance measures [29]. The LASSO, SVM-RFE, and random forest were implemented by “glmnet”, “e1071”, and “randomForest” packages in R, respectively. The key DELMRGs for AD were ultimately considered to be the common genes determined by all algorithms.

### Immune cell infiltration analysis

The relative levels of 28 immune cells in every sample of the GSE122063 dataset were quantified using the “ssgsea” algorithm from the “GSVA” R package [30]. Charoentong et al. conducted a previous study, from which a reference gene set of 28 immune cells was derived [31]. First, based on normality results, variances of immune cells between groups were calculated using either t-tests or Mann-Whitney Wilcoxon tests. Subsequently, the correlations between key DELMRGs and immune cells in AD were calculated via either Pearson correlation analyses or Spearman correlation analyses based on normality results.

### Verifying key DELMRGs in internal and external AD datasets

The expression variation of key DELMRGs between AD and control samples was initially evaluated in frontal cortex and temporal cortex tissues in GSE122063. In addition, to increase the reliability of key DELMRGs, the differences in relative levels of key DELMRGs between AD and controls were validated via two additional AD datasets (GSE132903 and GSE33000). Gene expression was normalized to the raw expression matrix using the z-score method. The comparisons were conducted using either a t-test or Mann‒Whitney Wilcoxon test based on normality assessment.

### Gene set enrichment analysis

First, for each DELMRG, a gene list was initially constructed for GSEA by arranging the genes in descending order of their correlation with the respective DELMRG. Subsequently, gene sets of KEGG from the MSigDB were utilized as references for GSEA [17]. Finally, GSEA was performed for each DELMRG by “clusterProfiler” R package [32]. Significantly enriched terms were visualized using the “GseaVis” R package [33].

### Construction of TF-miRNA regulatory networks for key DELMRGs

Potential miRNAs regulating the key DELMRGs were predicted using the miRDB database [34]. Additionally, potential TFs regulating the key DELMRGs were predicted based on JASPAR in the NetworkAnalyst 3.0 database [35, 36]. Finally, a miRNA-TF regulatory network for the key DELMRGs was visualized using Cytoscape software [37].

### Subcluster analysis with key DELMRGs

Based on the key DELMRGs, AD samples from GSE33000 were divided into two subclusters via “ConsensusClusterPlus” R package [38]. Subsequently, GSEA was performed to determine whether the “Alzheimer’s” KEGG pathway was significantly altered between the two subclusters [32]. In addition, the key DELMRG levels in two subclusters were compared. Furthermore, 28 immune cell levels were identified in two subclusters by the “ssgsea” method. Finally, the KEGG and HALLMARK pathways were evaluated in the two subclusters using the “GSVA” algorithm, and the top 10 most significantly altered pathways were visualized by heatmaps [30].

### Identification of immune-related genes interacting with key DELMRGs

The potential interactive targets of key DELMRGs in AD brain tissue were further investigated. First, 1793 IRGs were derived via the ImmPort database [39]. Subsequently, the correlation between each key DELMRG and each IRG was calculated in the GSE122063 dataset and GSE33000 dataset, and strongly relevant IRGs were identified (*P* < 0.05). Next, the top 500 genes exhibiting interactions with each DELMRG were identified from the STRING database [40]. Finally, the intersection of the correlation results and the PPI results were identified as potential interactive IRGs of key DELMRGs by Venn diagrams.

## Results

### Identification of DELMRGs

**Fig. 2:**
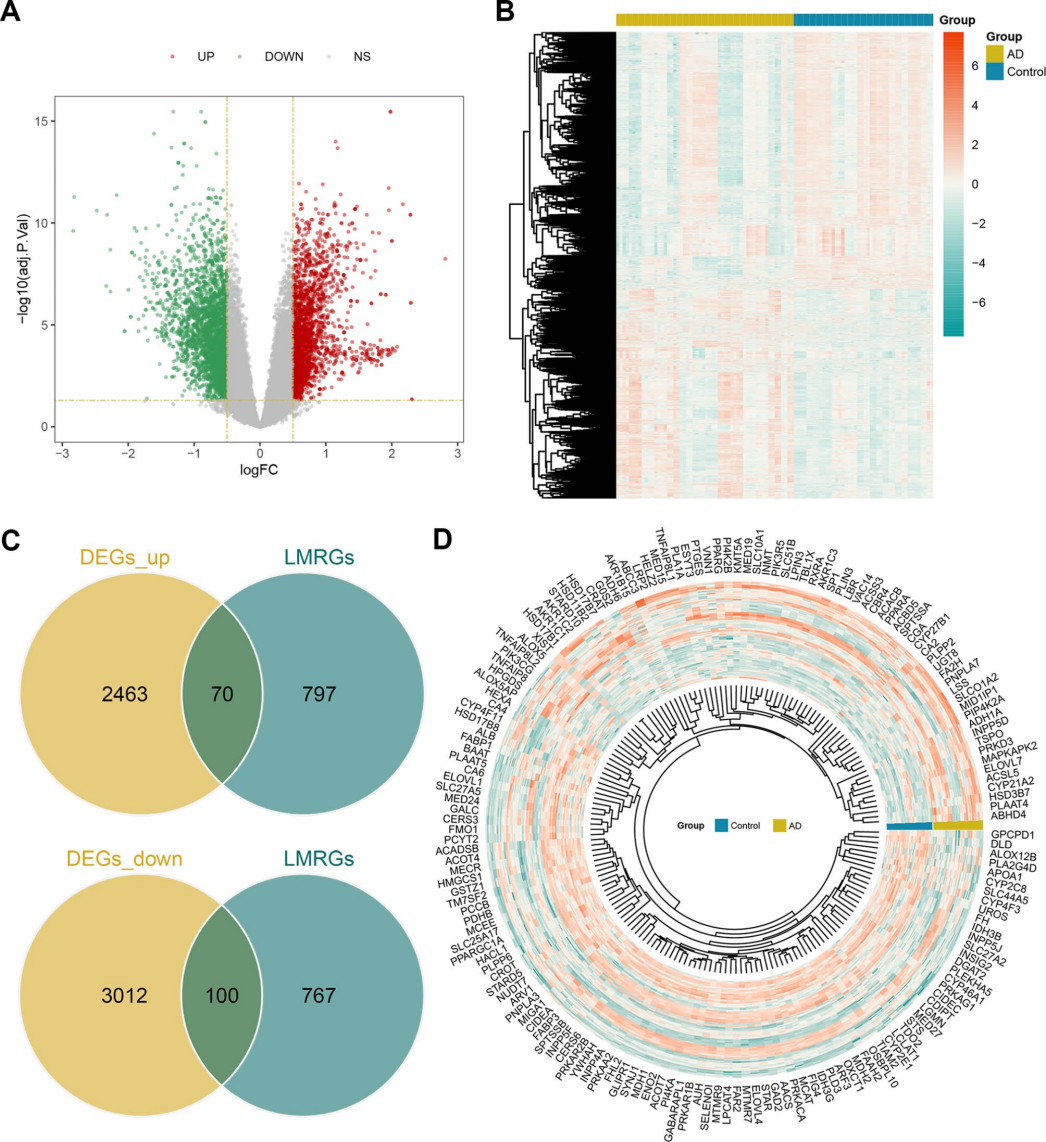
Identification of DELMRGs in AD. **(A)** Volcano map showing 5645 DEGs in GSE122063. **(B)** Heatmap showing 5645 DEGs in GSE122063. **(C)** Venn diagram identifying 70 up-regulated DELMRGs and 100 down-regulated DELMRGs. **(D)** Circular heatmap of 170 DELMRGs.

In GSE122063, 5645 DEGs were identified. Of these DEGs, 2533 exhibited upregulation, while 3112 showed downregulation (Fig. 2A and B). Subsequently, 170 DELMRGs were identified. Out of these, 70 exhibited upregulation, and 100 showed downregulation (Fig. 2C and D).

### Enrichment analyses of DELMRGs

**Fig. 3:**
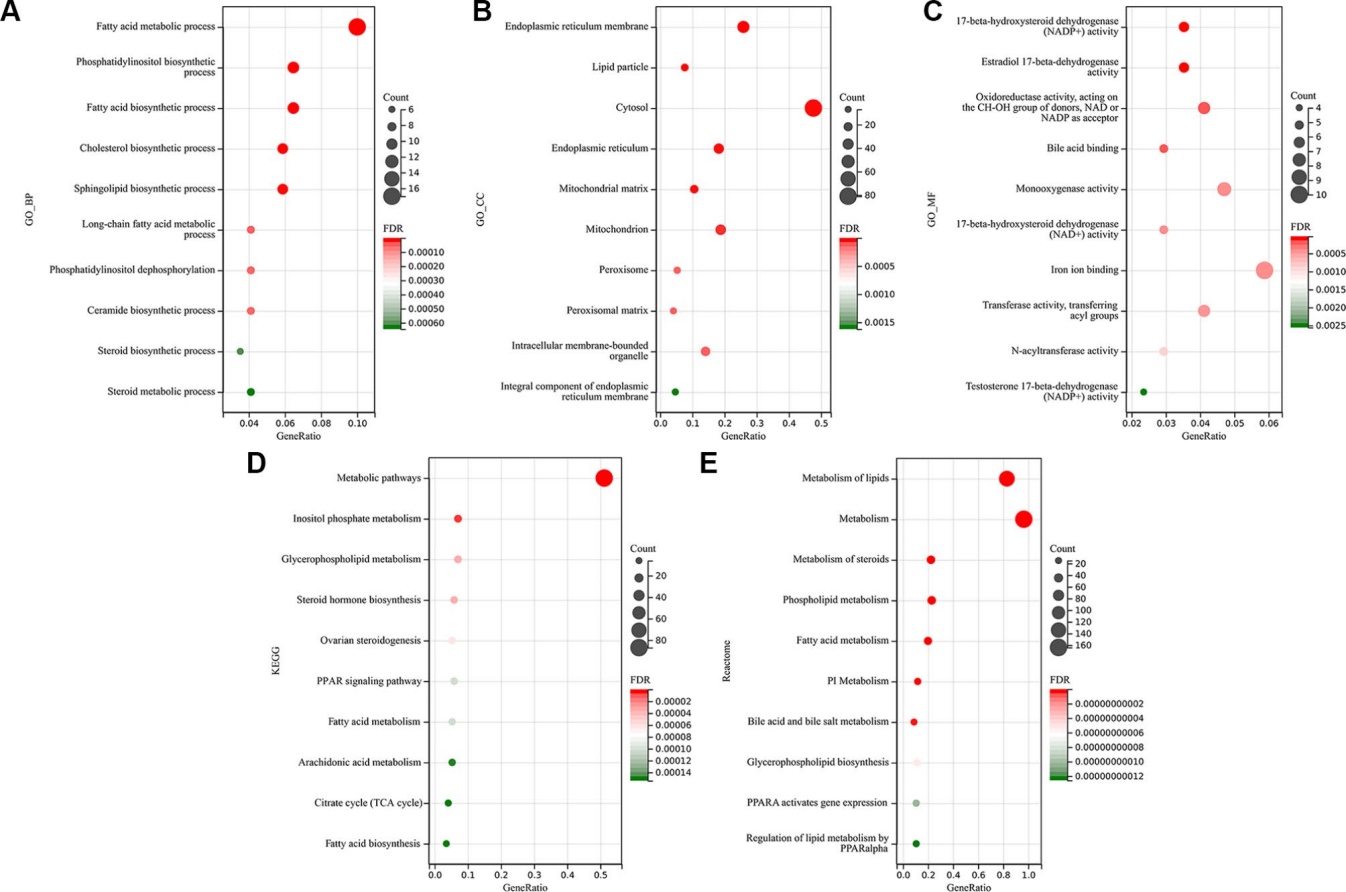
Analysis of functional enrichment in DELMRGs. Top 10 enriched terms for **(A)** GO_BP, **(B)** GO_CC, **(C)** GO_MF, **(D)** KEGG pathway, and **(E)** Reactome pathway analyses

Figure 3 presents the top 10 terms for each category. In the subcategory of GO biological process, DELMRGs were enriched in “fatty acid metabolic process”, “phosphatidylinositol biosynthetic process”, and “fatty acid biosynthetic process” (Fig. 3A). In the GO cellular component subcategory, DELMRGs were mainly enriched in “endoplasmic reticulum membrane”, “lipid particle”, and “cytosol” (Fig. 3B). In the GO molecular function subcategory, DELMRGs were mainly enriched in “17-beta-hydroxysteroid dehydrogenase (NADP+) activity”, “estradiol 17-beta-dehydrogenase activity”, and “oxidoreductase activity, acting on the CH-OH group of donors, NAD or NADP as acceptor” (Fig. 3C). KEGG analysis indicated that DEMLRGs were related to “metabolic pathways”, “inositol phosphate metabolism”, and “glycerophospholipid metabolism” (Fig. 3D). Reactome analysis suggested that DELMRGs were significantly enriched in “metabolism of lipids”, “metabolism”, and “metabolism of steroids” (Fig. 3E).

### Identification of AD key DELMRGs

**Fig. 4:**
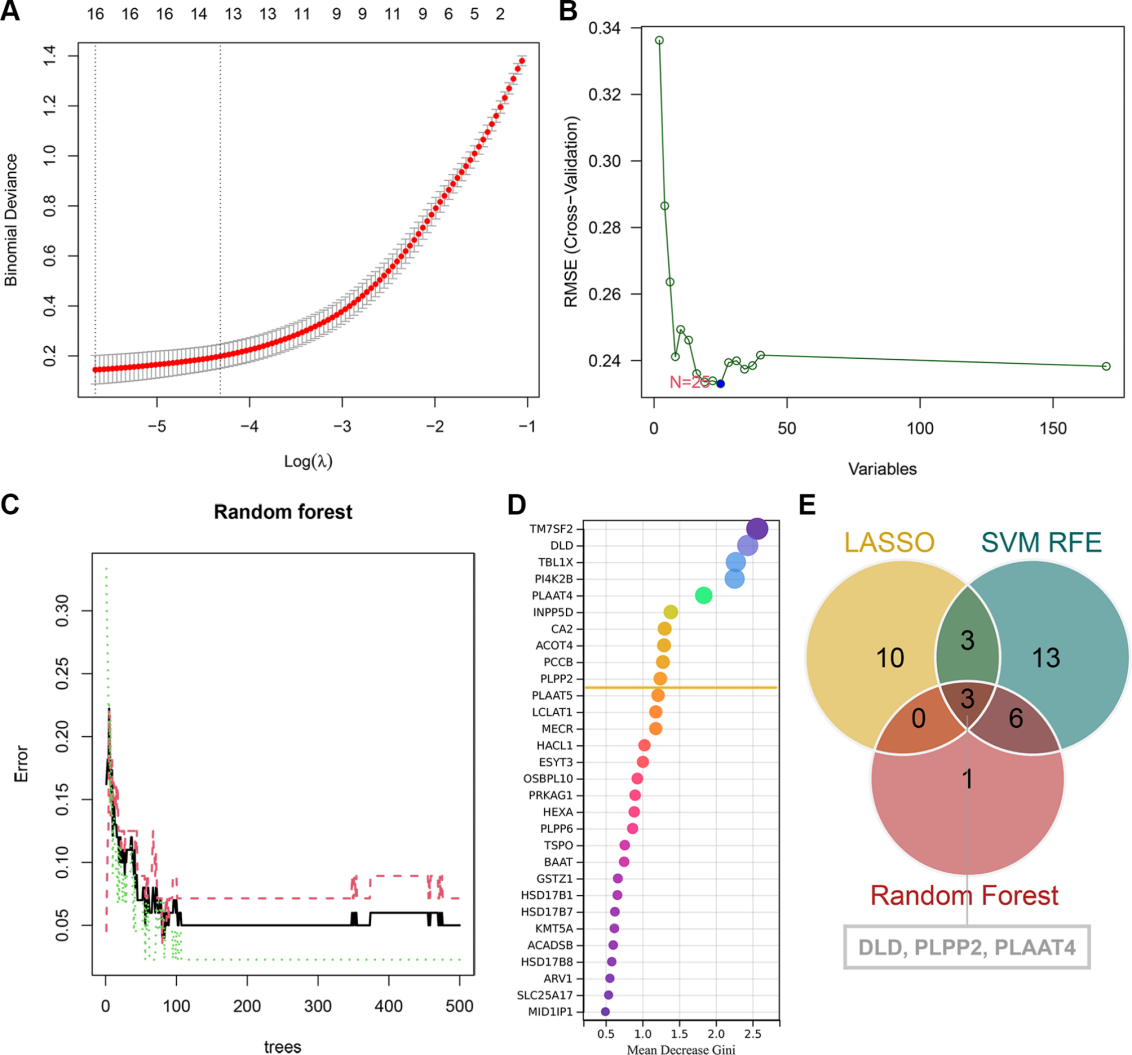
Identification of key DELMRGs. **(A)** LASSO regression yielded a total of sixteen gene signatures. **(B)** Using SVM- RFE, a total of twenty-five gene signatures were obtained **(C)-(D)** Random forest was used to extract top ten gene signatures. **(E)** The Venn diagram identified three key DELMRGs shared by three algorithms

LASSO regression analysis identified 16 gene signatures of 170 DELMRGs (Fig. 4A). SVM-RFE identified 25 gene signatures from 170 DELMRGs at the minimum RMSE (Fig. 4B). Random forest identified the top 10 gene signatures of 170 DELMRGs (Fig. 4C and D). Subsequently, the Venn diagram identified the three intersecting genes (DLD, PLPP2, and PLAAT4) (Fig. 4E). Therefore, DLD, PLPP2, and PLAAT4 might be the key DELMRGs involved in AD progression.

### Immune infiltration features of AD

**Fig. 5:**
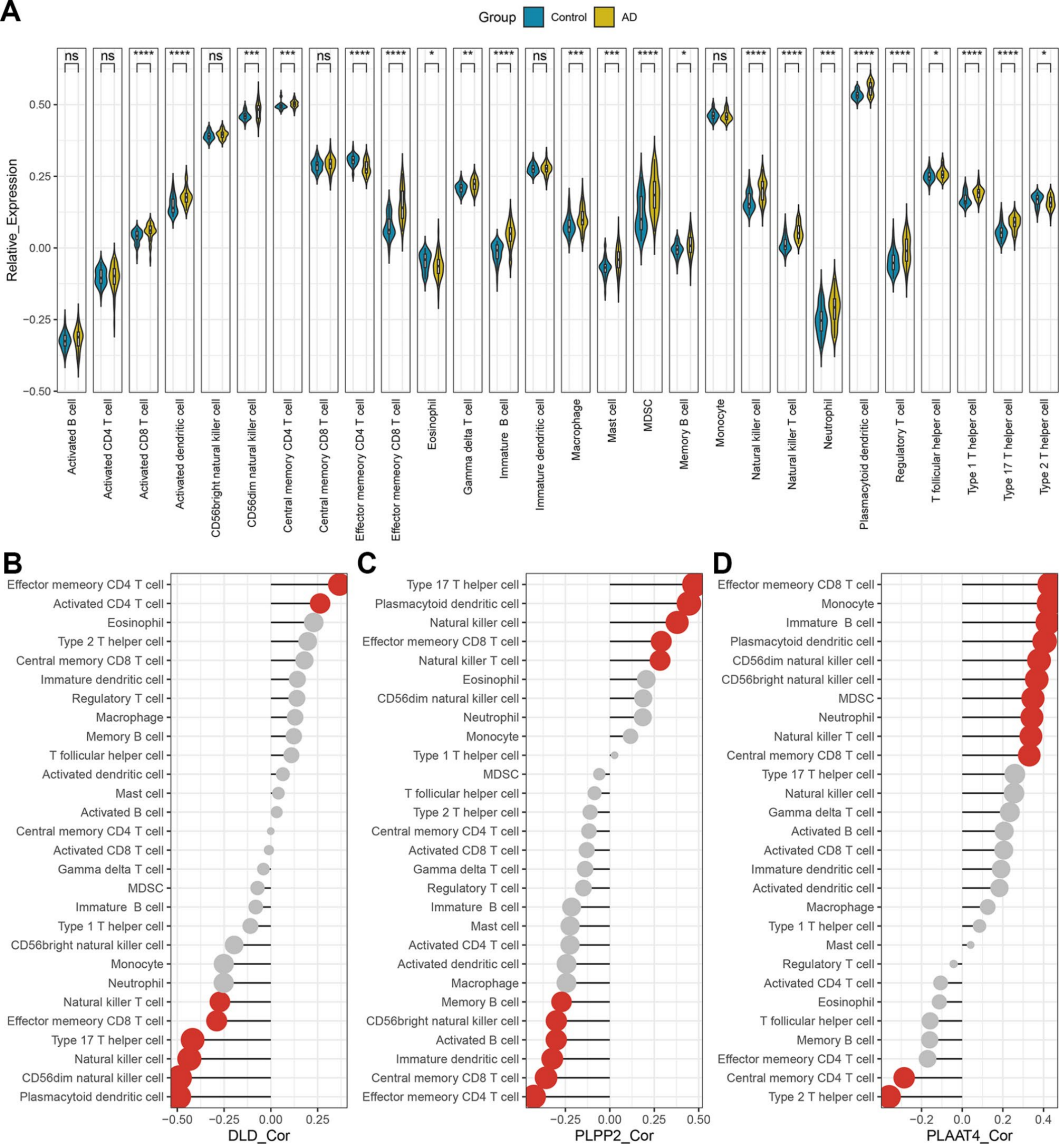
Immune infiltration results. **(A)** Comparison of 28 immune cell levels between AD and controls in GSE122063. **(B)** Correlation between DLD and diverse immune cells. **(C)** Correlation between PLPP2 and diverse immune cells. **(D)** Correlation between PLAAT4 and diverse immune cells

The expression pattern of immune cells differed distinctly between groups (Fig. 5A). Compared to those in controls, activated CD8 T cells, activated dendritic cells, effector memory CD8 T cells, immature B cells, MDSCs, natural killer cells, natural killer T cells, plasmacytoid dendritic cells, regulatory T cells, type 1 T helper cells, and type 17 T helper cells were significantly elevated in AD, while effector memory CD4 T-cell levels were significantly decreased (Fig. 5A). Figure 5B, C and D indicate the correlation of DLD, PLPP2, and PLAAT4, respectively, with each immune cell in the AD samples. Interestingly, effector memory CD8 T cells, natural killer T cells, and plasmacytoid dendritic cells were simultaneously correlated with all three DELMRGs.

### Validation of key DELMRGs

**Fig. 6:**
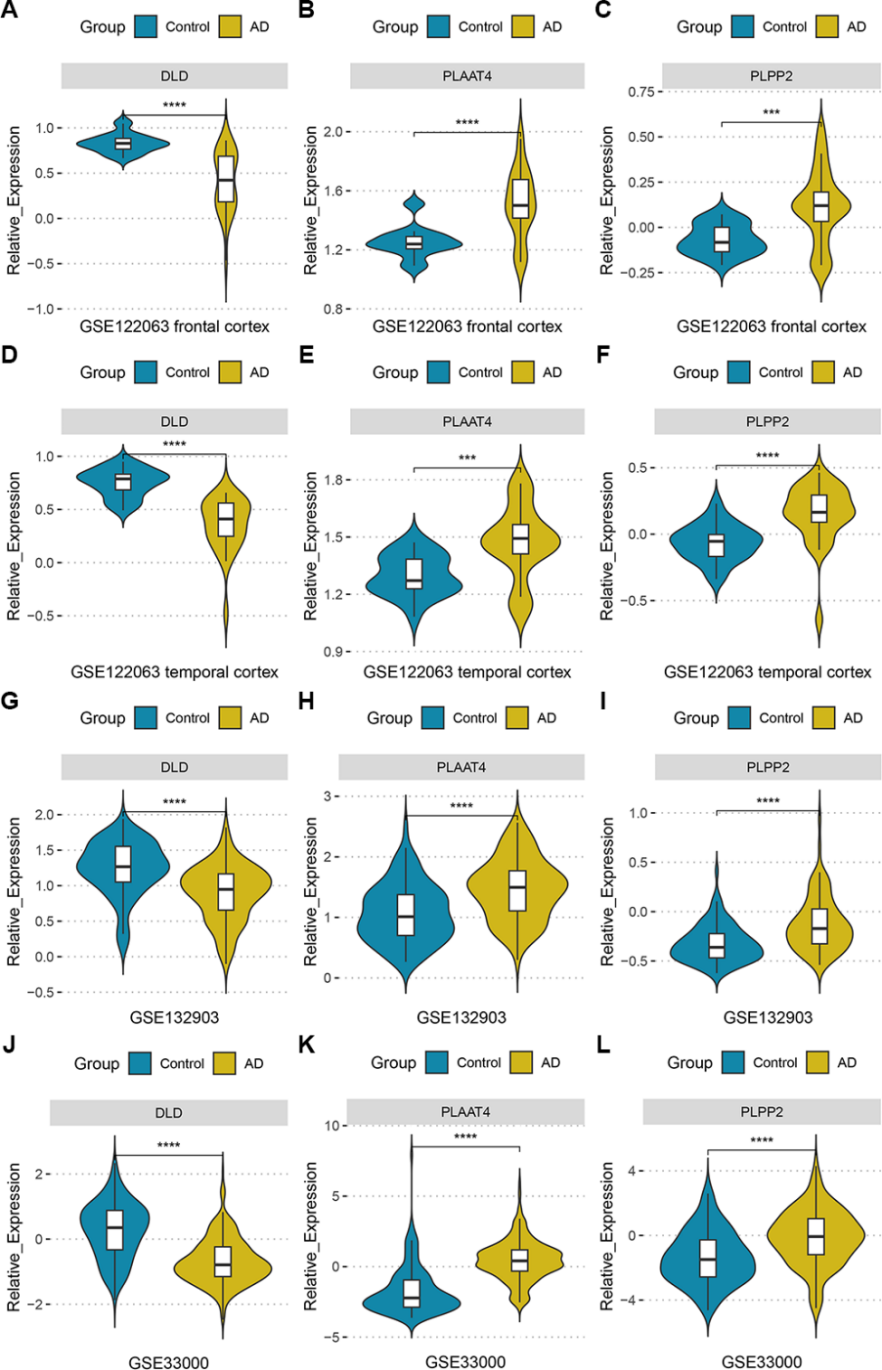
The three key DELMRGs expression levels in internal and external AD datasets. Gene expression was normalized to the raw expression matrix using the z-score method. **(A) – (C)** In frontal cortex samples of the GSE122063 dataset, DLD was down-regulated and PLAAT4 and PLPP2 exhibited upregulation in AD compared to controls. **(D) – (F)** In temporal cortex samples of the GSE122063 dataset, DLD was down-regulated and PLAAT4 and PLPP2 exhibited upregulation in AD compared to controls. **(G) – (I)** In the GSE132903 dataset, DLD was down-regulated and PLAAT4 and PLPP2 exhibited upregulation in AD compared to controls. **(J) - (L)** In the GSE33000 dataset, DLD was down-regulated and PLAAT4 and PLPP2 exhibited upregulation in AD compared to controls

Three key DELMRGs were validated in the internal dataset (two different cortical sites) and two additional AD datasets. In the GSE122063 dataset, DLD was down-regulated and PLAAT4 and PLPP2 were up-regulated in frontal and temporal cortex in AD (Fig. 6A-F). Likewise, in the GSE132903 dataset, compared to those in the control group, DLD was significantly decreased (Fig. 6G) and PLAAT4 (Fig. 6H) and PLPP2 (Fig. 6I) were significantly increased in AD. Finally, the same results were observed in the GSE33000 dataset (Fig. 6J-L). These consistent results indicate the potential of DLD, PLPP2, and PLAAT4 as key DELMRGs involved in AD progression.

### GSEA reveals the potential function of key DELMRGs in AD progression

**Fig. 7:**
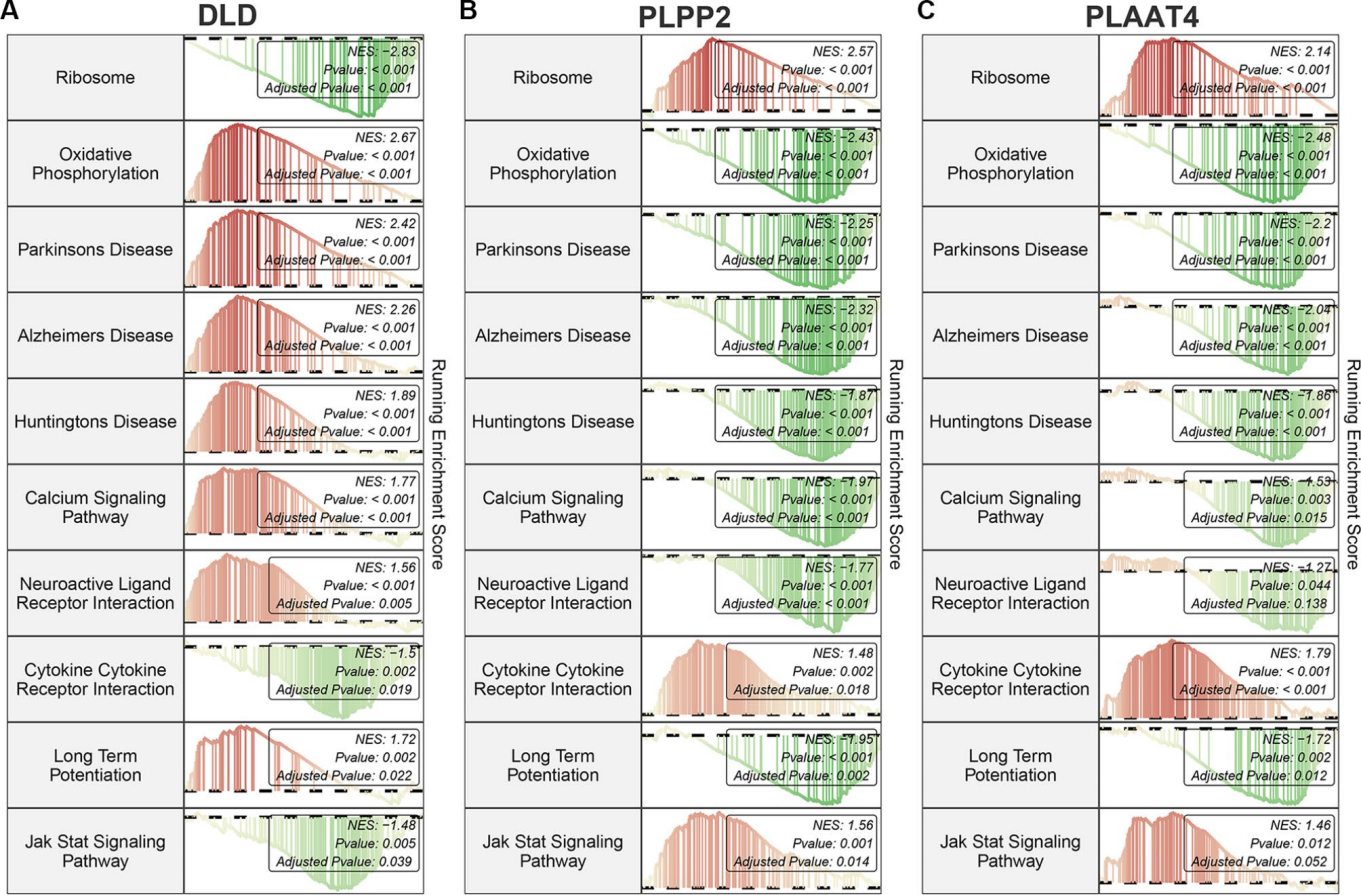
The potential interacting pathways of key DELMRGs identified by GSEA. **(A)** Correlation of DLD with common significantly enriched pathways. **(B)** Correlation of PLPP2 with common significantly enriched pathways. **(C)** Correlation of PLAAT4 with common significantly enriched pathways

The GSEA results indicated that DLD, PLPP2, and PLAAT4 were significantly associated with several KEGG terms simultaneously. For instance, DLD was positively correlated with “oxidative phosphorylation”, “Parkinson’s disease”, “Alzheimer’s disease”, “Huntington’s disease”, “calcium signaling pathway”, “neuroactive ligand receptor interaction”, and “long term potentiation” (Fig. 7A), while PLPP2 and PLAAT4 were negatively associated with these terms (Fig. 7B and C). Additionally, DLD was negatively related to “ribosome”, “cytokine cytokine receptor interaction”, and the “JAK STAT signaling pathway” (Fig. 7A), while PLPP2 and PLAAT4 were positively associated with these terms (Fig. 7B and C).

### TF-miRNA regulatory networks of key DELMRGs

**Fig. 8:**
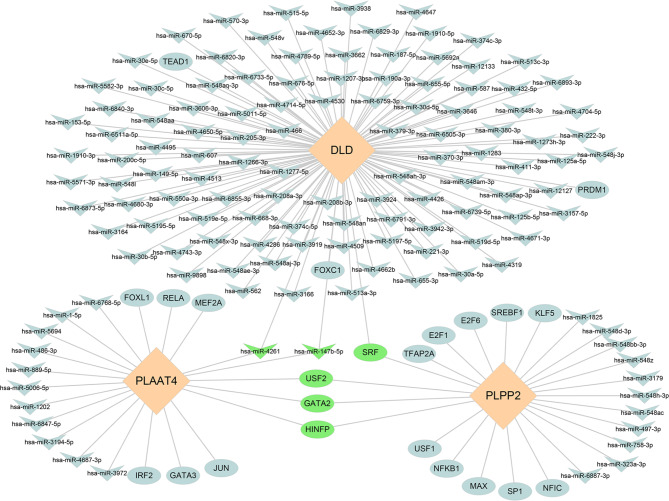
TF-miRNA regulatory networks of key DELMRGs.

A TF-miRNA regulatory network, including 23 TFs, 132 miRNAs, and 3 key DELMRGs, was constructed using the miRDB and NetworkAnalyst 3.0 databases (Fig. 8). Notably, miR-147b-5p and miR-4261 appear to potentially regulate both DLD and PLAAT4 concurrently. Additionally, SRF might exert simultaneous modulatory effects on DLD and PLPP2. Furthermore, USF2, GATA2, and HINFP might collectively regulate both PLPP2 and PLAAT4.

### Consensus clustering analysis of key DELMRGs

**Fig. 9:**
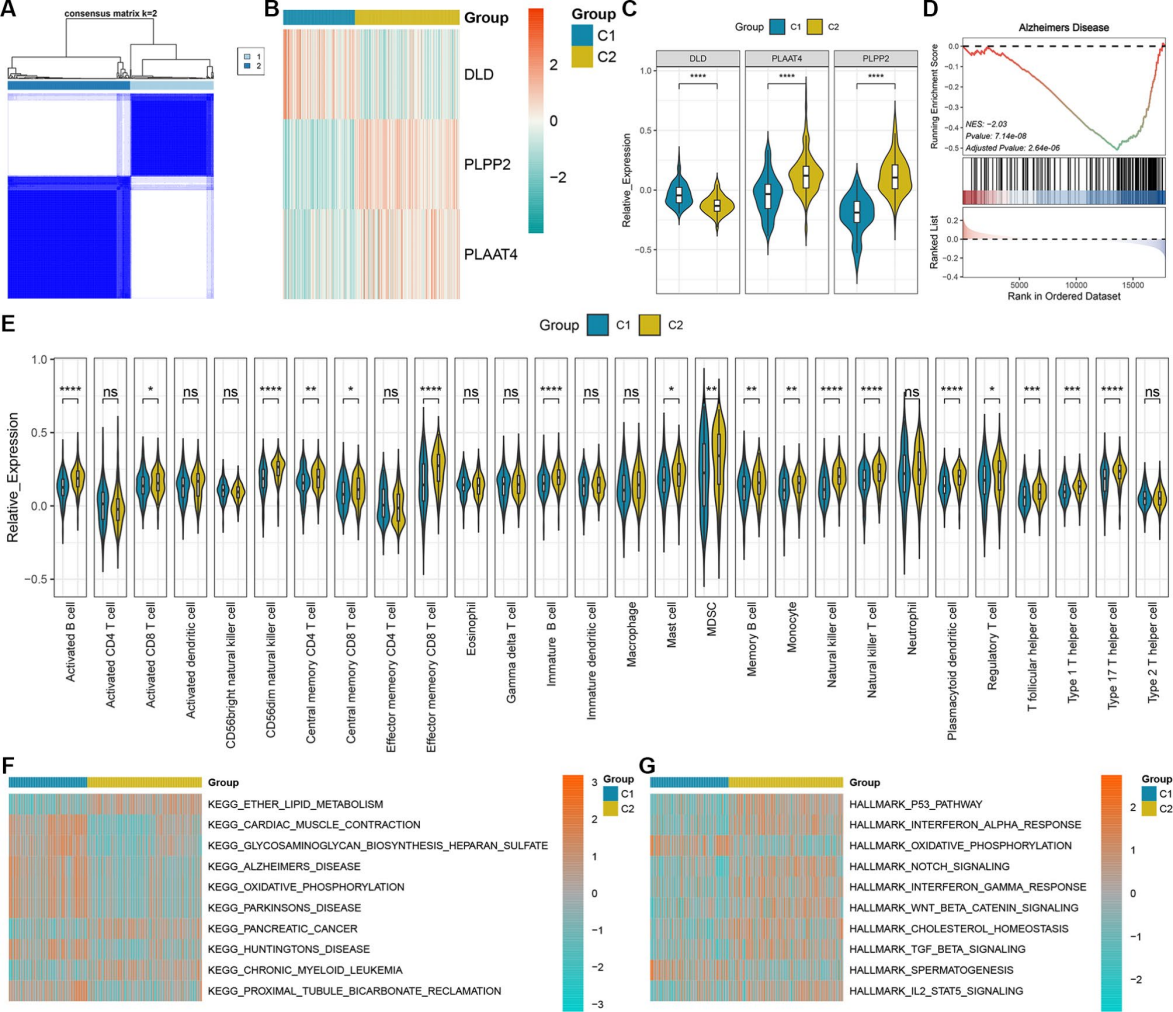
Identification of key DELMRG subtypes in AD samples. **(A)** Subclusters were constructed with key DELMRGs. **(B)** Heatmap of key DELMRG levels in two subclusters. **(C)** Violin plots of differences in key DELMRG levels between two subclusters. **(D)** GSEA performed among the two subclusters indicated that the AD KEGG term was significantly enriched. **(E)** Violin plots of variance of immune cell levels among two subclusters. **(F)** Top 10 significantly differentiated KEGG pathways among two subclusters. **(G)** Top 10 significantly differentiated HALLMARK pathways among two subclusters

The ConsensusClusterPlus algorithm separated the AD samples in GSE33000 into two subclusters based on three key DELMRGs (Fig. 9A). Heatmap and violin plots indicated that compared to cluster 1, DLD levels were decreased and PLPP2 and PLAAT4 levels were elevated in cluster 2 (Fig. 9B and C). GSEA showed that “Alzheimer’s disease” was significantly enriched, suggesting that the two subclusters identified by key DELMRGs represented distinct AD progressions (Fig. 9D). The 28 immune cell patterns of the two subclusters exhibited significant variation (Fig. 9E). In addition, Fig. 9F and G illustrate the top 10 KEGG and HALLMARK terms that exhibited significant distinctions between the two subclusters, respectively.

### Identification of IRGs interacting with key DELMRGs in AD

Ten IRGs interacting with DLD were identified. Of these IRGs, 6 were positively correlated with DLD in AD (GPI, IREB2, PDK1, PSMC1, PSMC2, and TUBB3), and 4 were negatively correlated with DLD in AD (CAT, CD4, PPARD, and RXRA) (Supplementary Figure S1). Twenty-five IRGs interacting with PLAAT4 were identified. Of these IRGs, 23 were positively correlated with PLAAT4 in AD (AKT1, B2M, BST2, HLA-B, HLA-DMA, HLA-DMB, HLA-DPA1, HLA-DRA, HLA-E, HLA-F, IFITM1, IRF7, IRF9, LTBP1, MAVS, OASL, PRDX1, PSMB8, S100A2, TAP1, TLR2, TLR3, and TNFAIP3), and 2 were negatively correlated with PLAAT4 in AD (CD8A and HSP90AB1) (Supplementary Figure S2). Two IRGs interacting with PLPP2 were identified. Specifically, PPARA showed a positive correlation with PLPP2, while GPI exhibited a negative correlation with PLPP2 (Supplementary Figure S3).

## Discussion

Lipidomic and metabolomic investigations have consistently demonstrated perturbations of diverse lipid classes that arise early in AD brains [10, 41]. Decades of research have uncovered intricate associations between lipid metabolism and the pivotal pathogenesis of AD, including amyloid plaque formation, oxidative stress, impaired energy production, inflammation in the brain, and deterioration of myelin [42]. Nevertheless, the complete understanding of the underlying molecular mechanisms remains uncertain. Through comprehensive bioinformatics analysis, 3 hub genes (DLD, PLPP2, and PLAAT4) linking lipid metabolism and AD were identified from transcriptomic data. Furthermore, the mechanisms leading to AD by these 3 key DELMRGs were investigated with immune infiltration analysis, GSEA, and a TF-miRNA regulatory network.

By comprehensive analysis, DLD, PLPP2, and PLAAT4 were determined as three key genes linking lipid metabolism and AD. Dihydrolipoamide dehydrogenase (DLD) is a crucial enzyme in eukaryotic energy metabolism. Changes in energy metabolism have been linked to AD progression [43], and thus, DLD is a potential therapeutic target. Variants of DLD was reported being involved in late-onset AD [44], and suppression of DLD has been found to attenuate Aβ toxicity [45]. Previous studies have identified decreased activity in enzyme complexes containing DLD in postmortem brain tissues of AD patients [46, 47]. Consistently, the present study found that DLD expression is significantly down-regulated in AD samples. PLPP2 is a gene that encodes phospholipid phosphatase 2 (PLPP2), a constituent of the phosphatidic acid phosphatase (PAP) group. PAPs transform phosphatidic acid into diacylglycerol, contributing to the generation of fresh glycerolipids and participating in receptor-triggered signal transmission facilitated by phospholipase D [48]. PLPP2 was reported being a contributing factor for late-onset AD risk [49]. It was shown that PLPP2 was significantly increased in early and late beta cells in AD [50], which is in line with the present results. PLAAT4 belongs to the phospholipase A and acyltransferase family, which possesses O- and N-acyltransferase activity and biosynthesizes N-acylated ethanolamine phospholipids [51]. It has been reported that PLAAT4 can interact with ribosomal protein lateral stalk subunit P0 (RPLP0), which affects the expression of various genes [52]. The downregulation of RPLP0 has been identified as a mediator of the endoplasmic reticulum stress reaction, which can trigger atypical autophagy [53]. Chen et al. identified up-regulated DNA methylation of RPLP0 in the brain tissues of AD, inhibiting gene expression [54]. Nevertheless, the complete comprehension of the role of PLAAT4 in AD remains unclear, necessitating additional investigation.

GSEA performed on gene lists arranged in descending order of correlation with key DELMRGs might provide insights into the potential functions of three key DELMRGs. The GSEA results demonstrated that these 3 key genes (DLD, PLPP2, and PLAAT4) were all associated with neurodegenerative diseases, which supports the notion that the identification of these key genes was accurate to some extent. In addition, GSEA results showed that DLD, PLPP2, and PLAAT4 were involved in ribosomes, oxidative phosphorylation, focal adhesion, calcium signaling pathways, and long-term potentiation. Recent findings indicate that malfunction of ribosomes could be critical for the pathology of AD [55]. During advanced stages of AD, changes in ribosomes and the process of protein synthesis in the cerebral cortex have been documented [56]. The build-up of defective amyloid precursor protein (APP) translation products, contributing to the characteristic traits of AD, has been linked to ribosome stalling in its etiology [57]. AD has been associated with mitochondrial malfunction and abnormalities in oxidative phosphorylation (OXPHOS). In AD brains, impaired OXPHOS leads to distinct mitochondrial dysfunction characterized by reduced ATP production, increased oxidative stress, and neuronal death [58]. Crucially, OXPHOS genes have been identified as a key pathway in AD using machine-learning techniques [59]. AD is linked to the pathophysiological mechanisms through the focal adhesion (FA) pathway, which integrates the physiological roles of amyloid precursor protein and tau [60]. FA proteins are responsible for transducing signals from outside cells to produce responses that include cytoskeletal changes. Fibrillar Aβ activates FA proteins, regulating cell cycle progression in AD [61]. The disruption of calcium balance affects the functioning of numerous G protein-coupled receptors (GPCRs) associated with AD, including interactions between neuroactive ligands and receptors. The pathology of AD is influenced by the dysregulation of GPCR signaling and calcium homeostasis [62]. Long-term potentiation (LTP) represents a mechanism of enduring memories at the cellular level. Studies have shown that synaptic plasticity, considered fundamental to learning and memory, could be compromised by exposure to Aβ in AD [63]. A decrease in basal synaptic strength and LTP deficits has been reported in AD [64]. Overall, these enriched pathways suggest that DLD, PLPP2, and PLAAT4 could be strongly related to AD progression.

AD is partly linked to neuroinflammation, which involves the participation of intrinsic immune cells, including microglia and astrocytes, and peripheral immune cells [65]. Therefore, immune infiltration analysis was further performed. The present results showed significantly diverse immune patterns among AD and controls. The presence of AD is linked to most categories of immune cells, in line with earlier findings that indicated the stimulation of both innate and adaptive immunity in individuals with AD [66, 67]. Three key DELMRGs were highly correlated with effector memory CD8 T cells and plasmacytoid dendritic cells. Although there was no significant correlation between the neuropathological hallmarks of AD and activated CD8 T cells, its elevation in CSF and peripheral blood has been reported to negatively impact AD cognitive symptoms [68]. Similarly, Gate et al. identified an increased effector memory CD8 T cells in both AD patients’ peripheral blood and CSF [69]. Plasmacytoid dendritic cells, which are a minority group of DCs, may potentially contribute to the coordination of immune responses and inflammation in AD [70]. Lai et al. identified that plasmacytoid dendritic cells are one of the most highly correlated immune cell types that can accurately predict AD progression [66]. In addition, AD samples from GSE33000 were further categorized into C1 and C2 subclusters using unsupervised clustering based on DLD, PLPP2, and PLAAT4. The 28 immune cell patterns of the two subclusters exhibited significant variation. Compared with the C1 subcluster group, patients in the C2 subcluster exhibited higher infiltration of immune cells. These findings indicate that these three key DELMRGs may contribute to AD morbidities by affecting the immune microenvironment.

In various complex diseases, regulatory biomolecules can act as potential biomarkers. A regulatory network of TF-miRNA consisting of 132 miRNAs and 23 TFs was identified in this study. The present findings reveal that miR-147b-5p potentially plays a role in regulating both DLD and PLAAT4. Interestingly, previous research has demonstrated that the T171C mutation, an APP variation, disrupts the regulatory impact of miR-147 on APP expression through the inhibition of miR-147 binding [71]. Among the interacting TFs, SRF simultaneously modulated DLD and PLPP2. SRF is expressed extensively in all cell types and contributes to the pathogenesis of various diseases, including cardiovascular diseases, nervous system diseases, and cancers [72]. The binding of SRF to the CArG DNA box, along with its interaction with various cofactors, regulates downstream genes [73]. In brain vascular cells, SRF and myocardin regulate Aβ clearance through the mediation of low-density lipoprotein receptor-related protein [74]. This implies that SRF and myocardin could potentially govern Aβ cerebrovascular clearance and influence AD progression [74]. Furthermore, the findings suggest that USF2, GATA2, and HINFP might collectively regulate both PLPP2 and PLAAT4. A meta-analysis suggested GATA2 as a common TF regulating mild cognitive impairment and AD [75]. The study by Gupta et al. revealed that CREB1 and HINFP are essential TFs participating in the crosstalk between AD and Parkinson’s disease, suggesting that targeting CREB1 and HINFP could be potential common therapeutic targets for both AD and PD [76].

Furthermore, the present study identified IRGs that were strongly correlated with the three DELMRGs in AD brain tissue samples by performing correlation analyses combining PPI analyses in two datasets. Ultimately, 10, 25, and 2 interacting IRGs that were strongly correlated with DLD, PLAAT4, and PLPP2, respectively, were identified. Future investigations on these targets may help uncover the intricate mechanisms of complex interactions between lipid homeostasis and the immune response involving key DELMRGs in AD progression.

### Strengths and limitations

The primary advantages of this study lie in the comprehensive bioinformatics analysis to pinpoint three hub genes (DLD, PLPP2, and PLAAT4) linking lipid metabolism and AD. This research offers insights into intricate associations between lipid metabolism and AD pathogenic mechanisms, supported by diverse methods, such as immune infiltration analysis, GSEA, and regulatory networks. The identified genes shed light on potential therapeutic targets and provide valuable insights for further research into AD pathogenesis. Nevertheless, this study has some limitations. Initially, although the bioinformatics analysis provided reliable evidence, whether the altered key genes serve as a causal factor or a consequential outcome, as well as the underlying mechanisms involved, remains for further experiments. Moreover, the immune infiltration assessment was conducted utilizing the pan-cancer immune cell gene set, and thus, the relevant results need to be interpreted with caution.

## Conclusion

Through a comprehensive analysis, the present study identified DLD, PLPP2, and PLAAT4 as key lipid metabolism-related genes that potentially contribute to AD progression. These findings underscore the important role of lipid metabolism dysfunction in AD pathogenesis, providing novel insights for AD prevention/treatment.

### Electronic supplementary material

Below is the link to the electronic supplementary material.

Supplementary Material 1

Supplementary Material 2

## Data Availability

The datasets examined in this research are accessible for download through the GEO database (https://www.ncbi.nlm.nih.gov/geo/). Detailed information is presented in Supplementary Table S1.

## Declarations

### Authors’ information

^1^Department of Anesthesiology, Third Xiangya Hospital, Central South University, Changsha, Hunan, 410013, China.

^2^Department of Nephrology, The Third Xiangya Hospital, Central South University, Changsha, Hunan, 410013, China.

## Abbreviations

AD: Alzheimer’s disease
CSF: cerebrospinal fluid
Aβ: β-amyloid
CNS: central nervous system
DEGs: differentially expressed genes
DELMRGs: differentially expressed lipid metabolism-related genes
FDR: false discovery rate
FA: focal adhesion
GEO: Gene Expression Omnibus
GO: Gene Ontology
GPCRs: G protein-coupled receptors
GSEA: gene set enrichment analysis
IRGs: immune-related genes
KEGG: Kyoto Encyclopedia of Genes and Genomes pathway
LASSO: least absolute shrinkage and selection operator
LMRGs: lipid metabolism-related genes
PPI: protein‒protein interaction
RMSE: root-mean-square error
SVM-RFE: support vector machine recursive feature elimination
TF: transcription factor
