# Supplementary material for: Identification of key lipid metabolism-related genes in Alzheimer’s disease
Source: Lipids Health Dis. 2023 Sep 22;22:155. doi: 10.1186/s12944-023-01918-9 (PMC10515010; doi:10.1186/s12944-023-01918-9)
Supplement: Supplementary file 1 — Supplementary Material 1 [file 12944_2023_1918_MOESM1_ESM.pdf]

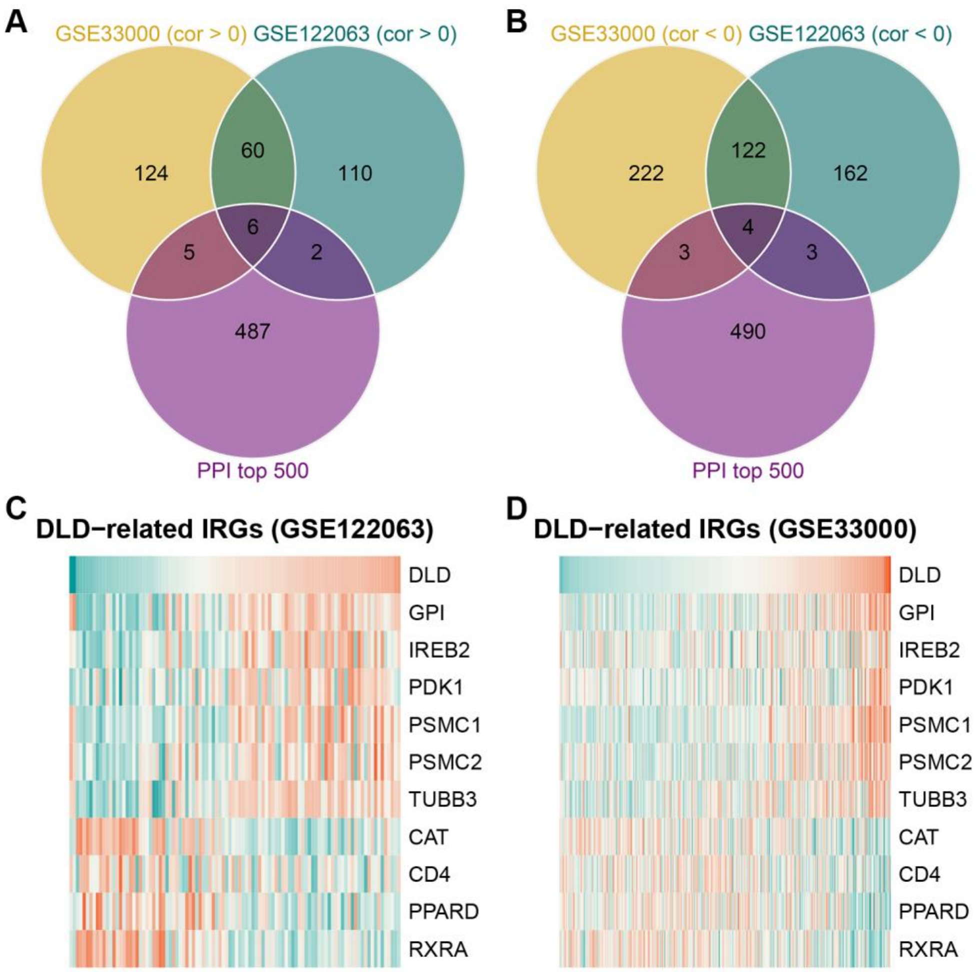

Supplemental Figure S1. Identification of IRGs interacting with DLD in AD. (A) Venn diagram identified 6 interacting IRGs positively correlated with DLD. (B) Venn diagram identified 4 interacting IRGs negatively correlated with DLD. (C) Heatmap of the relative expression levels of 10 IRGs interacting with DLD in GSE122063. (D) Heatmap of the relative expression levels of 10 IRGs interacting with DLD in GSE33000.

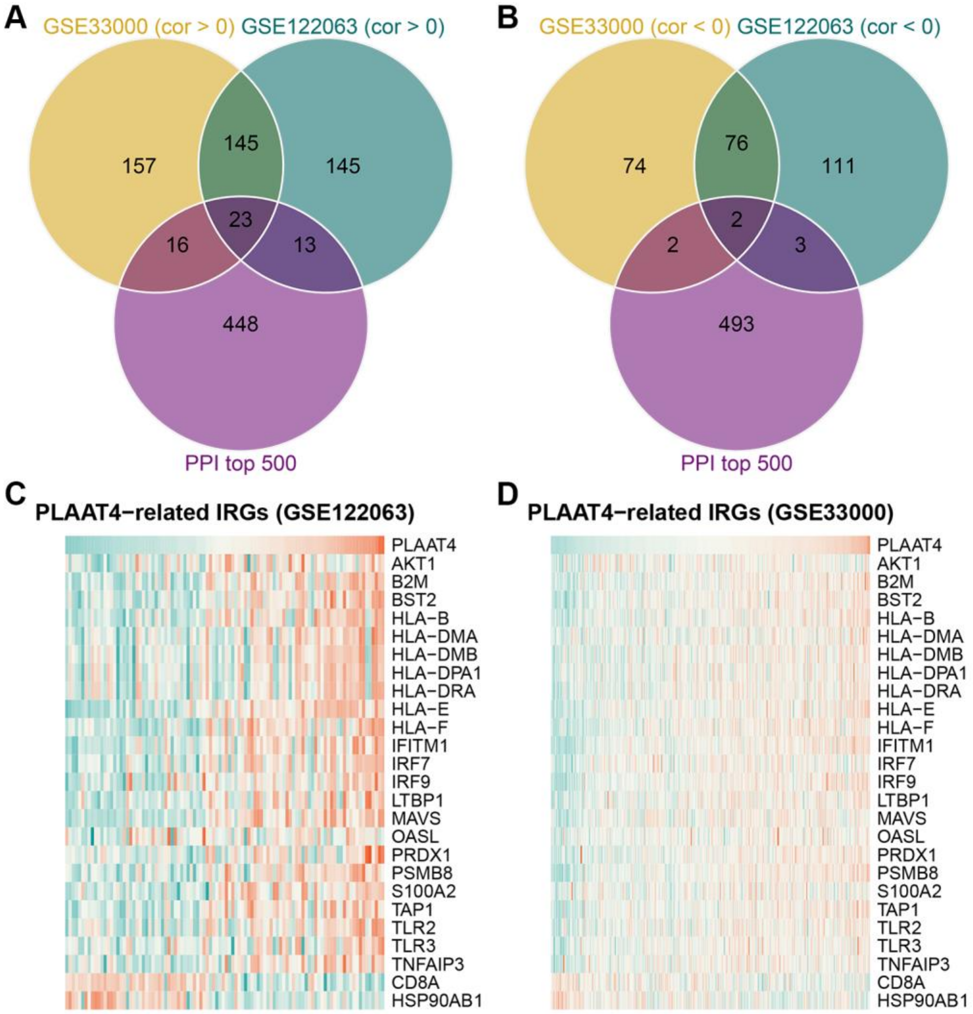

Supplemental Figure S2. Identification of IRGs interacting with PLAAT4 in AD. (A) Venn diagram identified 23 interacting IRGs positively correlated with PLAAT4. (B) Venn diagram identified 2 interacting IRGs negatively correlated with PLAAT4. (C) Heatmap of the relative expression levels of 25 IRGs interacting with PLAAT4 in GSE122063. (D) Heatmap of the relative expression levels of 25 IRGs interacting with PLAAT4 in GSE33000.

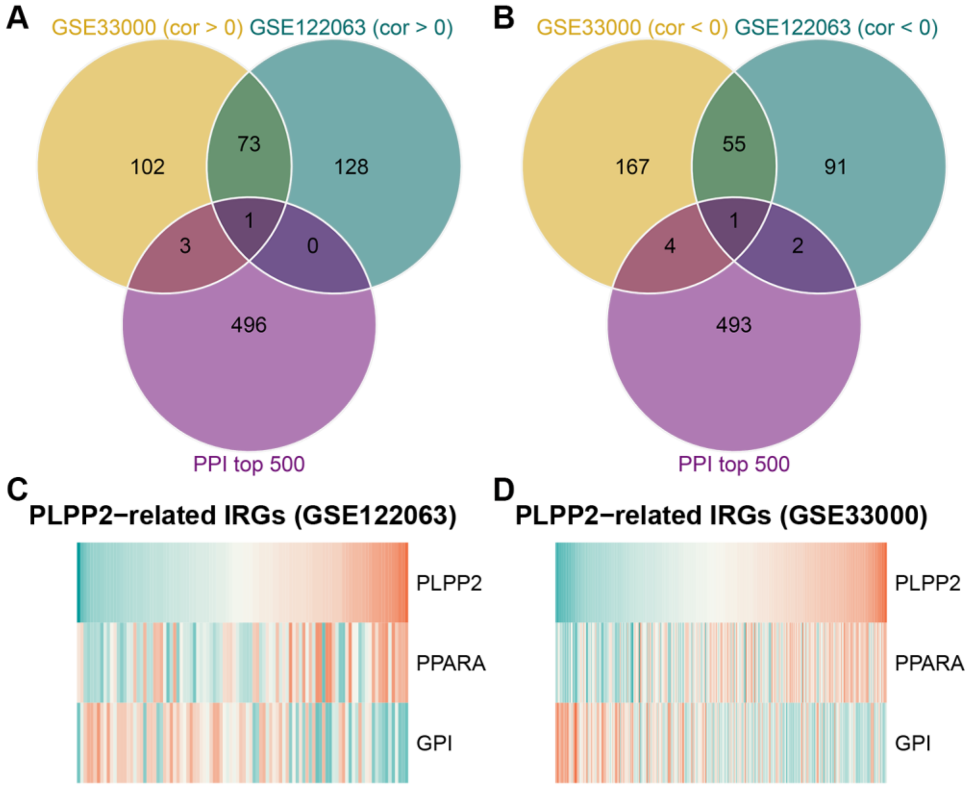

Supplemental Figure S3. Identification of IRGs interacting with PLPP2 in AD. (A) Venn diagram identified 1 interacting IRGs positively correlated with PLPP2. (B) Venn diagram identified 1 interacting IRGs negatively correlated with PLPP2. (C) Heatmap of the relative expression levels of 2 IRGs interacting with PLPP2 in GSE122063. (D) Heatmap of the relative expression levels of 2 IRGs interacting with PLPP2 in GSE33000.
